# Differentiation of Apricots of Different Geographic Origin in Central and Southern Europe by Applying ^87^Sr/^86^Sr Analysis: Potential and Limitations

**DOI:** 10.3390/foods11152239

**Published:** 2022-07-27

**Authors:** Micha Horacek, Lenka Klcova, Martina Hudcovicova, Katarina Ondreickova, Jozef Gubis, Stefan Hölzl

**Affiliations:** 1 HBLFA Francisco-Josephinum, BLT Wieselburg, Rottenhauserstr. 1, 3250 Wieselburg, Austria; 2 HBLA & BA of Pomology and Enology, Wienerstr. 74, 3400 Klosterneuburg, Austria; 3 Department of Lithospheric Research, Vienna University, Althanstr. 14, 1090 Vienna, Austria; 4 National Agricultural and Food Centre, Research Institute of Plant Production, Bratislavská Cesta 122, 921 68 Piestany, Slovakia; lenka.klcova@nppc.sk (L.K.); martina.hudcovicova@nppc.sk (M.H.); katarina.ondreickova@nppc.sk (K.O.); jozef.gubis@nppc.sk (J.G.); 5 RiesKraterMuseum, 86720 Noerdlingen, Germany; s.h@lmu.de; 6 Staatliche Naturwissenschaftliche Sammlungen Bayerns, Menzinger Straße 71, 80638 Munich, Germany

**Keywords:** provenance, strontium isotopes, bedrock geology, trace element, food fraud, TIMS

## Abstract

Consumers prefer food commodities of certain origins over the same products of other provenances and are willing to pay higher prices for them. Thus, it is possible to increase profit simply by giving an incorrect geographic origin to a product. To effectively control the declared geographic origin of food, the product itself has to be investigated to discover whether it actually originates from the declared origin, or if it has been mislabeled. Conventionally, control of a geographic origin is conducted by stable isotope analysis of the main elements, which has proven to be successful in numerous cases, but often reference data have to be produced anew for every harvest to control, resulting in additional costs and delays. Applying entirely geogenic parameters for the control of provenance requires reference data to be produced only once. As they do not vary between years and harvests, they can often be used for different (food) commodities. Here, we investigate whether the geographic origin of apricot samples can be controlled by their ^87^Sr/^86^Sr ratios measured by TIMS. The results show that Slovak and Hungarian apricots can be differentiated from the Wachau apricots, a well-known regional Austrian brand, and those from other regions in Austria, even though the differentiation from the latter is only partial. ^87^Sr/^86^Sr investigations can be a very potent tool; however, its success depends on the exact question that needs to be answered.

## 1. Introduction

Food authenticity describes the sum of good practices and the absence of bad practices. It means that the food commodity is pure, without unwanted additions, not adulterated, produced according to the respective codes of practice, and correctly labelled. Our study deals with one of the labelled parameters: the provenance, or geographic origin.

The indication of geographic origin has become an important parameter for food. On the one hand, consumers are increasingly aware of the relevance of geographic origin, as this might be a parameter for food quality (e.g., due to different rules in production and different environmental conditions), an important socio-economic factor (to protect regional economies and/or regional traditions), a safety issue (backtracing in the case of unconformities), or a legal requirement (protection from fraud or illegal activities).

Conventionally, the geographic origin of food is controlled by accompanying paperwork, but the latter is too unreliable, as intentional deception usually includes incorrect, faked, exchanged or counterfeited documents. Therefore, it is necessary to control the geographic origin by investigating the product itself. The applied methods often depend on the commodity being investigated and include a stable isotope analysis of the main elements of biogenic materials (H, C, N, O, S), isotope analysis of heavy elements such as Sr or Pb, element concentrations, molecular markers, organic compound concentrations, and screening methods such as FTIR, NMR, untargeted metabolomics, etc. (e.g., [1,2,3,4,5,6,7,8,9,10,11,12,13,14,15,16,17,18,19,20,21,22,23,24,25]). For food, usually, the method of choice is the isotope analysis of the main elements, of which numerous studies have already been published (e.g., [1,2,3,4,5,6,7,8,9,10], among many others). However, in many cases, this method relies on databases containing the results of numerous authentic reference samples, to which unknown or questioned samples are compared. This is necessary because the results may vary between and within seasons and vintages and might also be influenced by differing agricultural practices. Investigation of the ^87^Sr/^86^Sr signal has an advantage in that the accurate reference values from the reference samples might not be required, as the Sr signal is incorporated from the geologic bedrock into the soil, and from the soil into plants (and eventually into animals or humans) without fractionation ([26,27]). In addition, the results obtained for one commodity can usually be used for other commodities from the same geographic origin, as different species incorporate Sr in the same way and without fractionation. Examples have shown that such investigations can be highly successful (e.g., [11,12,13,14]). However, other examples have also documented that this method has its limitations (e.g., [15,16,17]) and the success or failure of this method depends on the conditions and circumstances, as well as on the exact problem that is being addressed ([17,18]). It should also be mentioned that the bioavailable Sr might possess a different isotope ratio than the bedrock ^87^Sr/^86^Sr. Therefore, it is important to investigate the reference samples, ideally of the same commodity as the one intended for control instead of the soil or bedrock samples, as the values of soil and bedrock might differ from the bioavailable ^87^Sr/^86^Sr ratios (e.g., [13,28]). 

Usually and traditionally, apricots are regional products, as they are perishable, and transport can further shorten their shelf-life as a consumable product. Thus, apricots are usually consumed regionally and, therefore, regional brands have been established. Some of these brands have obtained national and international relevance. However, in today’s world, many perishable goods are transported and are available globally. Therefore, control of the correct declaration of geographic origin needs to be established for delicate food commodities such as apricots, as consumers are willing to pay significantly higher prices for an especially valued provenance. Control of the declared geographic origin ensures protection for consumers against deception, for producers against dishonest competitors and for markets/traders from being liable for selling products under incorrect claims and labels. 

In Austria, the “Wachauer Marille” (Marille = apricot) is a regional, high-value apricot brand connected to the Wachau Valley, which is a part of the Danube River Valley that cuts through the Bohemian Metamorphic Massif. Besides being the origin of the Wachauer Marille, it is also well known as a wine region. The Wachauer Marille is an EU-registered product of protected origin (PDO: protected denomination of origin), and only apricots from 21 communities (Aggsbach-Markt, Albrechtsberg, Bergern im Dunkelsteinerwald, Droß, Dürnstein, Furth, Gedersdorf, Krems, Maria Laach, Mautern, Mühldorf, Paudorf, Rohrendorf bei Krems, Rossatz-Arnsdorf, Senftenberg, Spitz, Stratzing, Weinziel am Wald, Weißenkirchen, Schönbühel-Aggsbach, and Emmersdorf) in and around the Wachau Valley may be sold under this brand. Only certain apricot cultivars may be sold as Wachauer Marille ([29]); the main cultivar is the “Klosterneuburger”, which is similar to (or even a synonym of) the cultivar “Ungarische Beste”. In Austria, apricots labelled as “Wachauer Marille” are sold at a higher price than apricots of other provenances, be it from a national or international geographic origin. In recent years, repeated rumors have occurred that apricots of other geographic origins were illegally sold as “Wachauer Marille” (e.g., [30,31]). To prevent consumers from being deceived, control of the geographic origin of the Wachauer Marille is required. A preliminary study on the control of the geographic origin of Wachauer Marille by stable isotope analysis of the main elements was published by Horacek in 2017 ([32]). Besides Horacek in 2017 ([32]), to our knowledge, this is the first study on the control of the geographic origin of apricots.

In the present study, we tested whether ^87^Sr/^86^Sr analysis enables the differentiation of various geographic origins and unequivocal determination of the certain provenance on apricots from Austria, Slovakia, and other production regions. Furthermore, we compared our results with the published results of other commodities, to evaluate whether or not the results can be used for other foods. 

## 2. Materials and Methods

**Figure 1:**
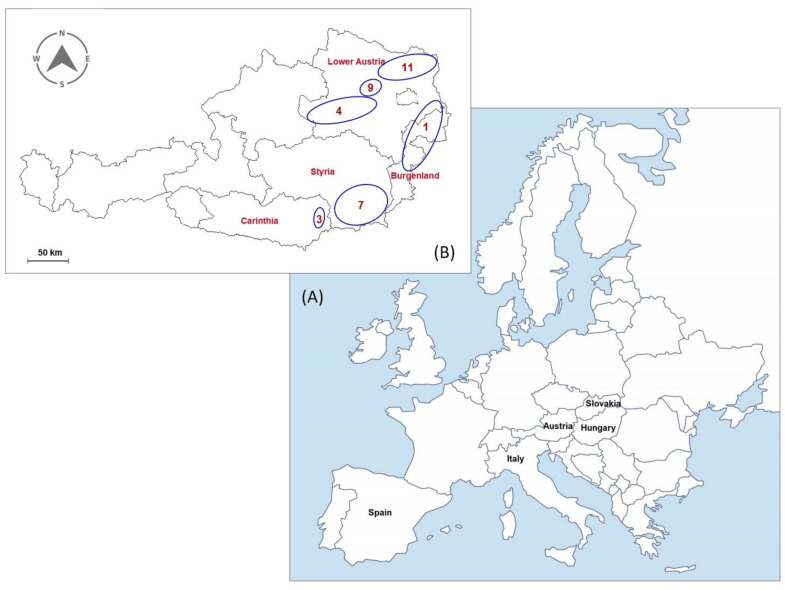
Maps showing countries (**A**) and Austrian regions in inset (**B**) of the geographic origin of investigated apricot samples. 1: Burgenland, 3: Carinthia, 4: Mostviertel, 7: Styria, 9: Wachau, 11: Weinviertel.

In 2019, 69 authentic apricot samples were collected directly from the apricot orchards of producers located in Austria, Slovakia and Hungary, where the apricots were collected from the trees. One commercial sample labelled as originating from “Wachau” (Wachau commercial), and two commercial samples of foreign origin were bought from supermarkets in Austria (Figure 1). For the control of reliability of the ^87^Sr/^86^Sr signal between vintages, 5 apricot samples from 2020 were analyzed: one commercial sample from Italy, one sample from Slovakia, two samples from the Wachau region and one sample from Weinviertel. A sample consists of at least 10 apricots, with a weight between 0.5 and 1 kg. The samples were either directly transferred or frozen until transfer to the laboratory, where the samples were stored in a freezer prior to sample preparation. The apricots (at least 10) from each sample were de-stoned, homogenized in a kitchen blender, and freeze-dried, after the supernatant juice was decanted. 

For Sr analysis, about 200 mg of the freeze-dried dry matter was ashed for two hours at 850 °C in quartz vessels. After dissolution of the ash in 6.5 N HNO_3_ and centrifugation, the Sr was purified and accumulated in vacuum columns filled with 0.05 mL of Sr-specific crown-ether resin (Sr-Spec^®^, Eichrom, Lisle, IL, USA). Strontium (estimated: 50 to 100 ng) was loaded on single-band Tungsten filaments (99.95% zone-refined, Cross, Moonachie, NJ, USA) in a mixture of TaCl_5_, HF, HNO_3_, H_3_PO_4_ and H_2_O. All relevant analytical steps were performed under clean room conditions using ultra-clean and blank-controlled reagents (sub-boiled in Dst-1000 stills, Savillex, Eden Prairie, MN, USA) and ultra-clean devices (qz-glass, PTFE, PFA, FEP, Nalgene, Savillex, etc.). The total analytical blank was included in the uncertainty consideration. Isotope ratios were analyzed in static mode (all relevant isotopes were simultaneously placed in Faraday cups) with a thermal ionization mass spectrometer (MAT261, Spectromat, Bremen, Germany) at the Ries Crater Museum in Nördlingen, Germany. Extant Rb was evaporated completely from the loaded filament by controlled pre-heating before the isotopic Sr composition was measured. Possible traces of ^87^Rb were corrected over the measured ^85^Rb/^86^Sr (<0.0001) and were included in the uncertainty consideration. The measured isotope values were normalized for mass fractionation using the naturally invariant value for ^88^Sr/^86^Sr of 8.37521 and the exponential fractionation law. Precision of the mass spectrometer runs was controlled by measuring the reference material SrCO_3_ NIST SRM 987. In the analysis period, 9 standard measurements resulted in a mean value of 0.710210 for ^87^Sr/^86^Sr, with a standard deviation (1SD) of 0.000024, 2 standard deviations of the mean (2SD_M) of 0.000016 and a 99% confidence interval of 0.000021. According to replicate analyses of natural samples and in-house standard materials, the accuracy of ^87^Sr/^86^Sr is estimated to be better than 0.0035% of the value.

Statistically significant differences among the geographic origins were tested using analysis of variance (ANOVA) and subsequently by using the “*post-hoc*” pairwise comparisons based on the Fisher’s least significant difference (LSD) procedure at the 95.0% confidence level. Additionally, a box plot displaying the dataset of ^87^Sr/^86^Sr values for each geographic origin and a frequency histogram showing a comparison of two samples were constructed using the software Statgraphics x64 (Statpoint Technologies, Inc., Warrenton, VA, USA). Similarly, this software was used to calculate a *t*-test to compare means, an F-test to compare standard deviations, a Mann–Whitney (Wilcoxon) W-test to compare medians, and a Kolmogorov–Smirnov (K-S) test to compare the distributions of the two samples.

## 3. Results

**Figure 2:**
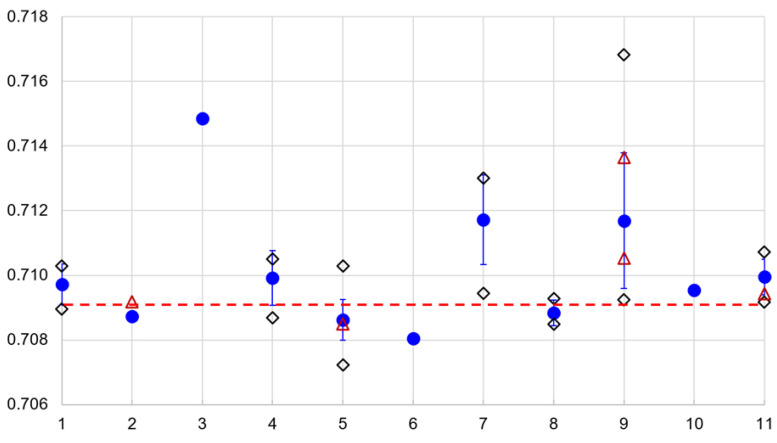
The ^87^Sr/^86^Sr-ratios of the investigated apricot samples from 2019 versus the regions of origin. *Y*-axis shows the ^87^Sr/^86^Sr ratio. The blue circles mark the average value, the bars show the standard deviation, the black diamonds indicate minimum and maximum values, and the red triangles represent the control samples from 2020. The red dashed line indicates the upper limit of marine ^87^Sr/^86^Sr ratios ([33]). 1: Burgenland, 2: Italy, 3: Carinthia, 4: Mostviertel, 5: Slovakia, 6: Spain, 7: Styria, 8: Hungary, 9: Wachau, 10: Wachau commercial, 11: Weinviertel.

From the 2019 vintage, the “Wachauer Marille” samples show an average ^87^Sr/^86^Sr ratio of 0.71169 with maximum and minimum values of 0.70925 and 0.71683, respectively; the Weinviertel samples show an average of 0.700995 with minimum and maximum values of 0.70917 and 0.71073, respectively; the Mostviertel samples show an average of 0.70992 with minimum and maximum values of 0.70896 and 0.71028, respectively; the Burgenland apricot samples have an average of 0.70972 with minimum and maximum values of 0.70896 and 0.71028, respectively; the Styrian samples show an average of 0.71172 with minimum and maximum values of 0.70944 and 0.71301, respectively; and one Carinthian sample has the value of 0.70872. The Slovakian samples show an average of 0.70863 with minimum and maximum values of 0.70868 and 0.71051, respectively, and the Hungarian samples show an average of 0.70884 with minimum and maximum values of 0.70848 and 0.70927, respectively. One commercial Italian sample has the value 0.70872, one commercial Spanish sample has the value of 0.70805, and the commercial Wachau apricot sample has the value of 0.70955 (Figure 2). From 2020, the commercial Italian sample has the value of 0.70918, the Slovak sample has the value of 0.70850, the Wachau samples have the values of 0.71053 and 9.71364, and the Weinviertel sample has the value of 0.70944 (Figure 2). 

A comparison of the results indicates that the Carinthian sample only overlaps with the Styrian and Wachau area results. The Hungarian samples are almost completely separated from the Wachau and Weinviertel samples. The Spanish sample only overlaps with the Slovakian results. The Italian sample only overlaps with the Slovakian, Styrian, Mostviertel and Hungarian samples. The commercial Wachau apricot sample is right at the limit of the standard deviation of the authentic Wachau samples. The samples from Burgenland, Mostviertel, Slovakia, Hungary and Weinviertel cluster together to form a group (Group 1) and possess a similar range of ^87^Sr/^86^Sr ratios, between approximately 0.7080 and 0.7110. The Carinthia, Styria and Wachau area samples show markedly higher values (Group 2), even though the groups of Styrian and Wachau apricot samples also include individuals possessing similar ratios as the group mentioned before. 

The ^87^Sr/^86^Sr ratios of the vintage 2020 apricot samples show excellent agreement with the results from 2019 (see Figure 2, red triangles). 

**Figure 3:**
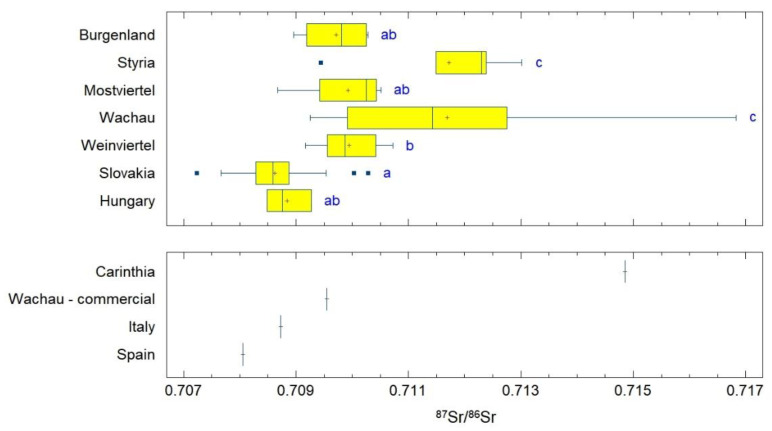
Box plots with statistical evaluation of the ^87^Sr/^86^Sr data of apricot samples from different geographic origins. The filled squares in the upper plot represent the outlier values. The different letters in the upper plot denote statistically significant differences among the geographic origin (LSD, α = 0.05). The lower part of the graph shows values for the geographic origin that contained only one apricot sample and were, therefore, not included in the statistical evaluation.

**Figure 4:**
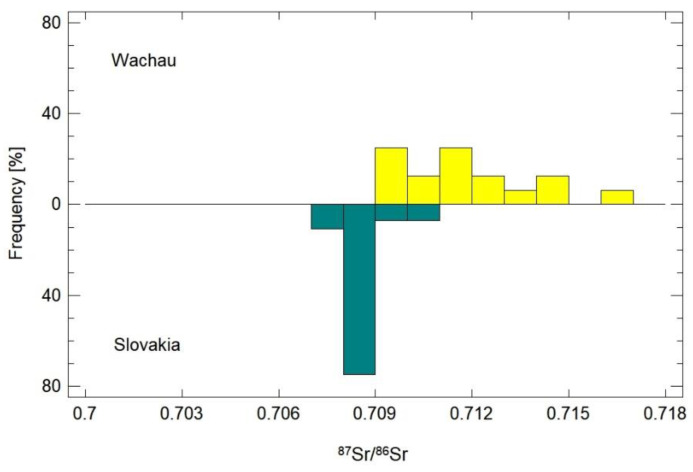
The frequency histogram displaying the comparison of values of ^87^Sr/^86^Sr from the apricot samples originating from Wachau and Slovakia. The height of each bar in the plot represents the percentage of observations in a single interval.

Statistical evaluation revealed that the Slovak samples are statistically significantly different from Weinviertel, Styria and Wachau. The Austrian samples from Styria and Wachau are not statistically different from each other, but they are different from all other samples (LSD, α = 0.05; Figure 3). The largest statistical difference was detected between Wachau and Slovakia. Therefore, the values of strontium from these two geographic origins were compared separately. Based on the histogram (Figure 4), 75% of the Slovak apricot samples showed a value in the range of 0.708–0.709. The samples from Wachau, in contrast to the Slovak ones, were more variable in strontium values and their profile is, therefore, milder and more gradual. A total of 25% of Wachau samples started at 0.709, and another 25% of the samples had a value between 0.711 and 0.712. The other 30% of samples from Wachau had a higher value, while strontium values from 0.712 were not measured in the Slovak samples. About 20% of the Slovak samples and about 38% of the Wachau samples had common values for strontium in the range of 0.709–0.711. Based on various comparisons of datasets from these two geographic origins, the following *p*-values were obtained: *t*-test *p* = 7.024 × 10^−9^; F-test *p* = 1.228 × 10^−7^; W-test *p* = 2.170 × 10^−7^; K-S test *p* = 1.782 × 10^−7^.

## 4. Discussion

The ^87^Sr/^86^Sr ratio of plant material is dominantly influenced by the ^87^Sr/^86^Sr ratio of the soil that the plant grows in. In 2022, Horacek ([17]) noted that there are indicators that the soil influence plays a more dominant role than the water (as the publication deals with milk samples, this statement should be even more valid for plants). Usually, the soil forms primarily from the underlying weathering bedrock, and thus the bedrock geology determines the plants’ ^87^Sr/^86^Sr ratio. Gregorcic et al., 2021, ([16]) postulated a differentiation by the ^87^Sr/^86^Sr ratio with the bedrock age; however, this attempt did not work well. As was shown by Horacek in 2022 ([17]), the type of bedrock is by far more relevant with respect to the ^87^Sr/^86^Sr ratio than its age. Marine carbonates usually show well-defined ^87^Sr/^86^Sr ratios in the range between 0.706 and 0.709 ([33]), depending on their age of deposition. Magmatic and metamorphic bedrocks, as well as siliciclastic deposits that emerged from those rocks, cover a much wider range of ^87^Sr/^86^Sr ratios, depending on their specific geological history (chemistry and age) from 0.704 to more than 0.720 ([17]).

Furthermore, in 2022, Horacek ([17]) noted that a heterogeneous bedrock geology consisting of different rock types blurs the potential of geographic differentiation by the ^87^Sr/^86^Sr ratio. This leads to very heterogeneous ^87^Sr/^86^Sr ratios within individual regions and a large overlap of ^87^Sr/^86^Sr ratios between regions. In addition, Goitom-Asfaha et al., 2011 ([18]), who investigated the geographic origin of wheat samples in Europe, stated that a classification according to bedrock geology and the use of the ^87^Sr/^86^Sr ratio can be problematic and less successful, due to oversimplified assumptions concerning bedrock geology. Overall, it can be said that a reasonable estimation of the Sr isotope ratios (and their variations) expected in a specific area is only possible if the geological and geographical conditions in the wider environment are thoroughly reviewed and taken into account.

The apricot origin regions summed up in Group 1 are mainly influenced by marine carbonate bedrock, but with a notable contribution of the higher ^87^Sr/^86^Sr ratio. This is most likely due to the co-existence of some siliciclastic material, as the ^87^Sr/^86^Sr ratio range exceeds marine carbonate values ([17]). Group 2 shows markedly higher ^87^Sr/^86^Sr ratios, but the large standard deviation of the Wachau and Styria samples indicates that several of the samples from these regions also possess rather low ^87^Sr/^86^Sr ratios, which means that these regions consist of a very heterogeneous geology (and thus a large range of ^87^Sr/^86^Sr ratios). The Carinthian sample has a high ^87^Sr/^86^Sr ratio. In Carinthia, there are only very few apricot orchards ([34]); this value might enable a good and unequivocal differentiation from the samples belonging to Group 1. Separation of the Slovak samples from the Wachau samples is also possible, see Figure 3 and Figure 4 (although not completely, as there is a very minor overlap), with the Slovak samples dominantly showing marine ^87^Sr/^86^Sr ratios, whereas all Wachau samples (besides one) have values beyond the marine range. One Italian sample and one Spanish sample both show ^87^Sr/^86^Sr ratios within the marine range, enabling a very good differentiation for the Spanish sample from all regions except Slovakia, and a good one for the Italian sample, which only overlaps with Mostviertel, Slovakia, Hungary, and Styria. However, Italy and Spain are both large countries, with each possessing a heterogeneous and diverse geology; therefore, these single values do not allow the conclusion that differentiation will be possible. This is evidenced, e.g., by Aguzzoni et al., 2020 ([15]), who documented how apples from Northern Italy already possess a wide range from very low to very high ^87^Sr/^86^Sr ratios. 

The control samples from the 2020 vintage provide good evidence that the ^87^Sr/^86^Sr ratio does not vary between years/vintages. This has already been stated in earlier articles dealing with other food commodities (e.g., [15]).

As the result of the Wachau commercial apricot sample lies well within the range of the authentic Wachau samples, there is no indication of an incorrect declaration of the geographic origin. 

Our results are in excellent agreement with the data of Swoboda et al., 2007 ([13]), who investigated the geographic origin of asparagus. Swoboda et al., 2007 ([13]), described a similar picture of differentiation of the geographic origin within Group 1, with slightly higher values of samples from the Marchfeld (the southeastern part of Weinviertel) with respect to samples from Slovakia and Hungary, but also documented some overlap of the ranges. 

## 5. Conclusions

By investigating the ^87^Sr/^86^Sr ratio of apricot samples from different regions in Austria and neighboring countries, our study achieved a differentiation of apricots from the Wachau region, which represents a well-known regional brand, and apricots from Slovakia and Hungary. Besides this, the apricot samples of most of the other regions overlap with regards to their ^87^Sr/^86^Sr ratios, as is to be expected due to the heterogeneous bedrock geology. Considering the range of individual regions defined by the standard deviation, further regions can be differentiated, for example, the Weinviertel apricot samples from the Slovak and Hungarian apricot samples. Still, some overlap occurs for many of the individual regions. Thus, the use of the ^87^Sr/^86^Sr ratio analysis for the control of the geographic origin of apricots from Austria/Central Europe is, in most cases, a challenge and does not enable a complete differentiation. Therefore, additional markers and methods are required for a complete and unequivocal differentiation/identification of the geographic origin. The ^87^Sr/^86^Sr ratio analysis can be a very potent tool for the discrimination or identification of the geographic origin; however, its success depends on the exact problem to be addressed and the question that needs to be answered. Heterogeneous bedrock geology in individual regions can be a problem for the differentiation of goods/samples originating from these regions by the ^87^Sr/^86^Sr ratio analysis, as it results in large variations within individual regions and, thus, overlapping results between the regions. Nevertheless, the successful separation of the apricot samples from the Wachau region with the apricots from Hungary and Slovakia demonstrates and evidences the relevance and potential of this method in well-defined and suitable settings. As the ^87^Sr/^86^Sr ratio patterns are usually independent of the commodity, which was confirmed by the results published for asparagus samples, the results should also be applicable for other (food) commodities (e.g., fruits and vegetables).

## Data Availability

Not applicable.

## References

[1] Camin F., Bontempo L., Heinrich K., Horacek M., Kelly S.D., Schicht C., Thomas F., Monahan F.J., Hoogewerff J., Rossmann A. (2007). Multi-element (H, C, N, S) stable isotope characteristics of lamb meat from different European regions. Anal. Bioanal. Chem..

[2] Camin F., Perini M., Bontempo L., Giongo L. (2009). Multi-Element (H, C, N, O) Stable Isotope Characterization of Blueberries. Acta Hortic..

[3] Camin F., Larcher R., Nicolini G., Bontempo L., Bertoldi D., Perini M., Schlicht C., Schellenberg A., Thomas F., Heinrich K. (2010). Isotopic and elemental data for tracing the origin of European olive oils. J. Agric. Food Chem..

[4] Cueni F., Nelson D.B., Boner M., Kahmen A. (2021). Using plant physiological stable oxygen isotope models to counter food fraud. Sci. Rep..

[5] Griboff J., Baroni M.V., Horacek M., Wunderlin D.A., Monferrán M.V. (2019). Multielemental and Isotopic Fingerprint Enables Linking Soil, Water, Forage and Milk Composition, Assessing the Geographical Origin of Argentinean Milk. Food Chem..

[6] Horacek M., Min J.-S. (2010). Discrimination of Korean beef from beef of other origin by stable isotope measurements. Food Chem..

[7] Horacek M., Min J.-S., Soja G. (2010). Discrimination between ginseng from Korea and China by light stable isotope analysis. Anal. Chim. Acta.

[8] Horacek M., Hansel-Hohl K., Burg K., Soja G., Okello-Anyanga W., Fluch S. (2015). Control of origin of sesame oil from various countries by stable isotope analysis and DNA based markers—A pilot study. PLoS ONE.

[9] Horacek M., Nives O., Magdas A., Wunderlin D., Sucur S., Maras V., Misurovic A., Eder R., Wyhlidal S., Cus F. (2021). Isotope analysis (13C, 18O) of wine from Central and Eastern Europe and Argentina, 2008 and 2009 vintages: Differentiation of origin, environmental indications and variations within the countries. Front. Sustain. Food Syst..

[10] Schellenberg A., Chmielus S., Schlicht C., Camin F., Perini M., Bontempo L., Heinrich K., Kelly S.D., Rossmann A., Thomas F. (2010). Multielement Stable Isotope Ratios (H, C, N, S) of Honey from different European Regions. Food Chem..

[11] Bontempo L., Larcher R., Camin F., Hölzl S., Rossmann A., Horn P., Nicolini G. (2011). Elemental and isotopic characterisation of typical Italian alpine cheeses. Int. Dairy J..

[12] Fitzpatrick R.M., Winkelman D.L., Johnson B.M. (2021). Using elemental and isotopic data to evaluate Esox lucius (Linnaeus, 1758) natal origins in a hydrologically complex river basin. Fishes.

[13] Swoboda S., Brunner M., Boulyga S.F., Galler P., Horacek M., Prohaska T. (2008). Identification of Marchfeld asparagus using Sr isotope ratio measurements by MC-ICP-MS. Anal. Bioanal. Chem..

[14] Victor V., Ross S., Karine P., André P., Jean-François H., David W. (2015). Strontium Isotope Characterization of Wines from the Quebec (Canada) Terroir. Procedia Earth Planet. Sci..

[15] Aguzzoni A., Bassi M., Pignotti E., Robatscher P., Scandellari F., Tirler W., Tagliavinia M. (2020). Sr isotope composition of Golden Delicious apples in Northern Italy reflects the soil 87Sr/86Sr ratio of the cultivation area. J. Sci. Food Agric..

[16] Gregorcic S.H., Ogrinc N., Frew R., Necemer M., Strojnik L., Zuliani T. (2021). The Provenance of Slovenian Milk Using 87Sr/86Sr Isotope Ratios. Foods.

[17] Horacek M. (2022). The Need to Consider Geochemistry When Interpreting Sr-Isotopes. Comment on Gregorčič et al. The Provenance of Slovenian Milk Using ^87^Sr/^86^Sr Isotope Ratios. Foods.

[18] Goitom Asfaha D., Quetel C.R., Thomas F., Horacek M., Wimmer B., Heiss G., Dekant C., Deters-Itzelsberger P., Hölzl S., Rummel S. (2011). Combining isotopic signatures of N(87Sr)/(86Sr) and light stable elements (C, N, O, S) with multi-elemental profiling for the authentication of provenance of European cereal samples. J. Cereal Sci..

[19] Đurđić S., Stanković V., Ražić S., Mutić J. (2021). Is a Lead Isotope Ratios in Wine Good Marker for Origin Assessment?. Front. Chem..

[20] Sharma S., Ambardar S., Bhagat N., Raj S., Trakroo D., Horacek M., Zouagui R., Sbabou L., Vakhlu J. (2021). Microbiome fingerprint as biomarker for geographical origin and heredity in Crocus sativus: A Feasibility Study. Front. Sustain. Food Syst..

[21] Horacek M., Hola M., Tobolkova B., Kolar K., Vaculovic T., Mikes O., Marosanovic B., Philipp C., Lojovic M., Polovka M. (2019). Investigation of geographic origin of wine from border regions: Results from investigation of two vintages. BIO Web Conf..

[22] Bagheri B., Philipp C., Horacek M., Bauer F., Setati M.E. (2019). Distinction of microbial diversity in grape must of different grape varieties and regions from Austria and South Africa using Automated Ribosomal Intergenic Spacer Analysis. BIO Web Conf..

[23] Zhou Y., Kim S.-Y., Lee J.S., Shin B.K., Seo J.-A., Kim Y.-S., Lee D.Y., Choi H.-K. (2021). Discrimination of the Geographical Origin of Soybeans Using NMR-Based Metabolomics. Foods.

[24] Gerginova D., Simova S., Popova M., Stefova M., Stanoeva J.P., Bankova V. (2020). NMR Profiling of North Macedonian and Bulgarian Honeys for Detection of Botanical and Geographical Origin. Molecules.

[25] Estekia M., Memarbashia N., Simal-Gandarab J. (2022). Classification and authentication of tea according to their geographical origin based on FT-IR fingerprinting using pattern recognition methods. J. Food Compos. Anal..

[26] Flockhart D.T., Kyser T.K., Chipley D., Miller N.G., Norris D.R. (2015). Experimental evidence shows no fractionation of strontium isotopes (87Sr/86Sr) among soil, plants, and herbivores: Implications for tracking wildlife and forensic science. Isot. Environ. Health Stud..

[27] Horn P., Schaaf P., Holbach B., Hölzl S., Eschnauer H. (1993). 87Sr/86Sr from rock and soil into vine and wine. Z. Lebensm. Unters. Forsch..

[28] Thomsen E., Andreasen R. (2019). Agricultural lime disturbs natural strontium isotope variations: Implications for provenance and migration studies. Sci. Adv..

[29] Amtsblatt der EU (2012/C 140/18, ff.). https://eur-lex.europa.eu/legal-content/DE/TXT/PDF/?uri=uriserv:OJ.C_.2012.140.01.0018.01.DEU.

[30] Sailer A. (2015). Durchbruch im Kampf gegen Plagiate. Kurier.

[31] Zahrl J. (2015). Mogelvorwurf bei Marillenverkauf. Kurier.

[32] Horacek M. (2017). Isotope investigation of apricots from the Wachau-area/Lower Austria (“Wachauer Marille”) to control the declared geographic origin: A pilot study—First results. Mitt. Klosterneubg..

[33] Zaky A.H., Brand U., Buhl D., Blamey N., Bitner M.A., Logan A., Gaspard D., Popov A. (2019). Strontium isotope geochemistry of modern and ancient archives: Tracer of secular change in ocean chemistry. Can. J. Earth Sci..

[34] https://www.statistik.at/web_de/statistiken/wirtschaft/land_und_forstwirtschaft/agrarstruktur_flaechen_ertraege/obst/116868.html.

